# Defining triple‐negative breast cancer with neuroendocrine differentiation (TNBC‐NED)

**DOI:** 10.1002/cjp2.318

**Published:** 2023-04-20

**Authors:** Sean M Hacking, Evgeny Yakirevich, Yihong Wang

**Affiliations:** ^1^ Laboratory Medicine Program University Health Network, Toronto General Hospital Toronto Canada; ^2^ Department of Pathology and Laboratory Medicine Rhode Island Hospital and Lifespan Medical Center Providence RI USA; ^3^ Department of Pathology and Laboratory Medicine Warren Alpert Medical School of Brown University Providence RI USA

**Keywords:** breast, neuroendocrine, carcinoma, *RB1*, *TP53*, TNBC‐NED

## Abstract

Primary breast neuroendocrine (NE) neoplasms are uncommon, and definitions harbor controversy. We retrospectively collected 73 triple‐negative breast cancers (TNBC) and evaluated NE biomarker expression along with p53 aberrant staining (which correlates with *TP53* gene mutation) and Rb protein loss by immunohistochemistry. In the study cohort, we found 11 (15%) cases of TNBC with neuroendocrine differentiation (TNBC‐NED) showing positivity for one or more NE markers (synaptophysin/chromogranin/insulinoma‐associated protein 1 [INSM1]). We also identified one separate small cell neuroendocrine carcinoma. Histologic types for these 11 TNBC‐NED cases were as follows: 8 invasive ductal carcinoma (IDC) not otherwise specified (NOS), 2 IDC with apocrine features, 1 IDC with solid papillary features. INSM1 had the highest positivity and was seen in all 11 carcinomas. Seven (64%) cases showed p53 aberrant staining, 6 (55%) had Rb protein loss, while 6 (55%) had p53/Rb co‐aberrant staining/protein loss. TNBC‐NED was associated with Rb protein loss (*p* < 0.001), as well as p53/Rb co‐aberrant staining/protein loss (*p* < 0.001). In 61 cases negative for NE markers, 37 (61%) showed p53 aberrant staining, while 5 (8%) had Rb protein loss. We also analyzed genomic and transcriptomic data from The Cancer Genome Atlas (TCGA) PanCancer Atlas of 171 basal/TNBC patients. Transcriptomic analysis revealed mRNA expression of *RB1* to be correlated negatively with *SYN1* mRNA expression (*p* = 0.0400) and *INSM1* mRNA expression (*p* = 0.0106) in this cohort. We would like to highlight the importance of these findings. TNBC‐NED is currently diagnosed as TNBC, and although it overlaps morphologically with TNBC without NED, the unique p53/Rb signature highlights a genetic overlap with NE carcinomas of the breast.

## Introduction

Primary breast carcinoma with neuroendocrine (NE) features is an uncommon tumor that was first recognized in the 1960s; argyrophilic tumor cells were first recognized in breast mucinous carcinomas from ultrastructural studies by Feyrter and Hartmann [1]. In the decades since, immunohistochemical markers such as chromogranin (CHGA) and synaptophysin (SYN), insulinoma‐associated protein 1 (INSM1), neuron‐specific enolase, and CD56 (N‐cellular adhesion molecule) have become widely used for the identification of the NE phenotype.

An oft‐deliberated topic in breast pathology is the identification and significance of NE differentiation (NED) in primary breast cancer. Compared with other sites such as the lung and gastrointestinal (GI)‐pancreatic systems, a cogent taxonomy remains largely elusive. The recently updated World Health Organization classification, fifth edition classifies invasive breast carcinomas (IBCs) with NED into neuroendocrine neoplasms (NENs) of the breast; tumors with >90% of cells showing histological evidence of NED, inclusive of both well‐differentiated neuroendocrine tumors (NETs) and poorly differentiated neuroendocrine carcinomas (NECs), along with IBC, no specific type (NST) with NE features (IBC‐NE) with ≤90% NED [2, 3]. Most breast NETs are ER positive, while solid papillary carcinomas and some mucinous carcinomas are well‐recognized breast cancer‐specific types that express NE markers.

Morphologically, NE features are not always well characterized in breast cancer. However, breast NETs can have spindled, plasmacytoid, polygonal or signet ring cells, often with granular, eosinophilic, clear, or finely vacuolated cytoplasm. Nuclei can often be pleomorphic with irregular nuclear membranes, while chromatin can be evenly distributed with inconspicuous nucleoli, hyperchromatic, or be vesicular with prominent nucleoli [4]. Breast NECs include small cell neuroendocrine carcinoma (SCNEC), characterized by densely packed hyperchromatic cells with scant cytoplasm, streaming, and crush artifact, along with large cell neuroendocrine carcinoma (LCNEC), with large cell size, polygonal shape, low nuclear‐cytoplasmic ratio, finely granular eosinophilic cytoplasm, and occasionally prominent nucleoli, and ambiguous small versus large cell morphology (ANEC), the latter being extremely rare.

The expression of NE markers can vary depending on anatomic site and degree of differentiation. Distinct NE markers for differentiation are currently used in different organ systems (e.g. only CHGA and SYN in the gastrointestinal tract and pancreas versus CHGA, SYN, and CD56 in the lung). Morphology suggestive of NED does not always reflect NE biomarker expression. CHGA is a glycoprotein secreted by neurons and NE cells and is sequestered in secretory granules [5]. The expression level of CHGA depends on the number of secretory granules, therefore poorly differentiated NEC may show only focal CHGA. SYN is a membrane protein of small vesicles present in the presynaptic vesicles in nerve terminals and NE cells [6]. In breast carcinomas, SYN and CHGA expression have been found to correlate with each other but not with CD56 [7]. INSM1 is a zinc finger transcription factor expressed transiently in embryonic NE tissue, thought to coordinate termination of cell division with differentiation of NE and neuroepithelial cells. In adult tissues, INSM1 has been identified in multiple tumors of NE or neuroepithelial origin. INSM1 was detected in 88.3% of NE and neuroepithelial neoplasms [8]. INSM1 expression showed similar clinicopathological and biomarker profiles to CHGA and SYN in a large IBC cohort using tissue microarrays (TMAs) by immunohistochemistry (IHC) [9]. INSM1 showed a sensitivity of 37%, more sensitive than CHGA (34%) and CD56 (16%) but less than SYN (95%) [9].

Combined *TP53* mutation and retinoblastoma protein (Rb) loss have been shown to be associated with NEC of the breast [10]. *TP53* mutations are the most common feature of triple‐negative breast cancers (TNBCs) but can also be seen in ER+ breast cancer. Rb loss is known to occur in breast cancer, and its significance remains largely unclear. However, previous studies have demonstrated Rb loss in TNBC to be significantly associated with AR expression, lower Nottingham grade, and metastatic disease to bone [11], further suggesting luminal‐like biology and the possibility of response to CDK4/6 inhibition.

As most NEC cases (7/13, 54%) were found to be triple‐negative in the study by Bean *et al* [10], we would like to share our findings of NE biomarker expression with p53 aberrant staining and Rb protein loss in a cohort of 73 TNBC cases. We would like to extend these findings providing further insights into TNBC with NED (TNBC‐NED), and answer whether p53 and Rb define the molecular portrait of these tumors.

## Materials and methods

### Case selection

Upon Lifespan Health System Institutional Review Board approval (Lifespan IRB: 751551‐10), a retrospective slide review for patients over the age 18 years was performed from February 2019 to June 2022. Seventy‐three TNBC cases were included and evaluated for NE biomarkers and p53 and Rb IHC protein expression. All methods were carried out in accordance with relevant guidelines and regulations and informed consent was not required by the Lifespan Health System Institutional review board secondary to the retrospective nature of this study. The Cancer Genome Atlas (TCGA) data were retrieved for TNBC cases from CBIO portal: https://www.cbioportal.org/study?id=62fbe1cbc381017d3f301945.

### Pathology examination

**Figure 1:**
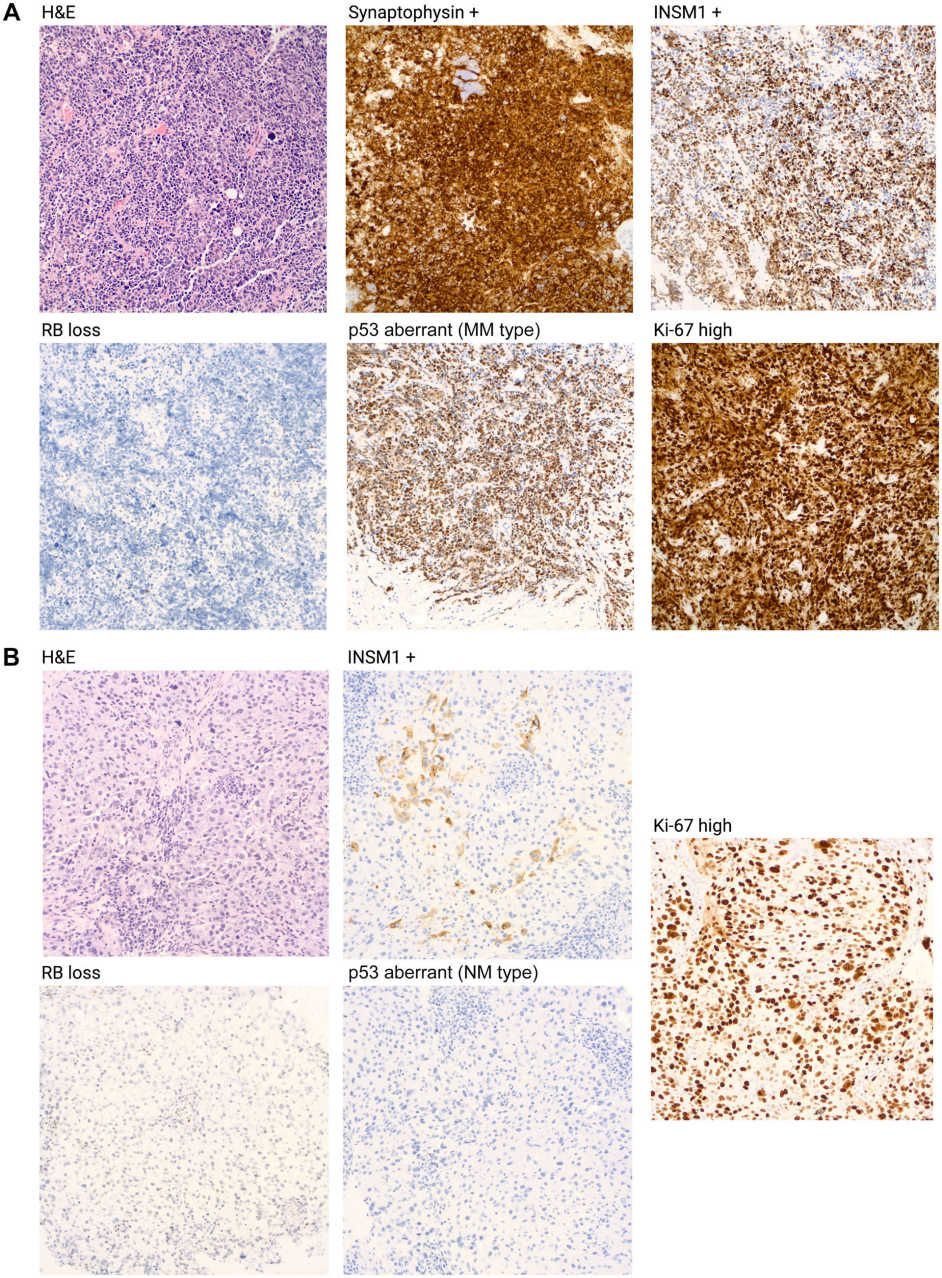
Histological and immunohistochemistry findings in breast cancers showing neuroendocrine differentiation. (A) 51‐year‐old patient with a poorly differentiated TNBC with small cell morphology and positive synaptophysin expression, showing Rb protein loss and p53 aberrant staining (MM type) by IHC with a high Ki‐67 proliferative index. The terminology for this tumor is NEC, but more specifically SCNEC. (B) 37‐year‐old with a poorly differentiated TNBC with positive INSM1 expression and a high Ki‐67 proliferative index found to have Rb protein loss and p53 aberrant staining (NM type) by IHC. We propose the terminology for this tumor as TNBC‐NED.

All cases were reviewed by two breast pathologists to identify any breast tumors with characteristic features, including prominent necrosis and mitosis, densely packed hyperchromatic cells with scant cytoplasm, streaming, and crush artifact for SCNEC, and large cell size, polygonal shape, low nuclear‐cytoplasmic ratio, finely granular eosinophilic cytoplasm, occasionally prominent nucleoli for LCNEC. We also evaluated some NET morphological features such as round and oval cell shape, plasmacytoid cytology, inconspicuous nucleoli, and ‘salt and pepper’ chromatin or architectural features such as solid nodules, nests, and trabecular with an organoid pattern. TNBC‐NED was supported by immunoreactivity with neuroendocrine IHC markers (SYN1, CHGA, and INSM). Figure 1 demonstrates histological and IHC findings from breast cancers showing NED, including both SCNEC and TNBC‐NED.

### Immunohistochemistry

Anti‐estrogen receptor (Dako, Santa Clara, CA, USA; clone 1D5), progesterone receptor (Dako; clone 1A6), HER2/neu (Dako; HercepTest), anti‐p63 (Biocare, Pacheco, CA, USA; clone 4A4), CHGA (Cell Marque, Darmstadt, Germany; clone LK2H10); SYN (Dako; clone DAK‐synap), INSM1 (Santa Cruz, CA, USA; clone A‐8); Rb (BD‐Science, Sane Jose, CA, USA; clone G3‐245O), and p53 (Agilent Technologies, Santa Clara, CA, USA; clone DO7) were used for IHC. Immunoreactivity was detected using the Dako EnVision method according to the manufacturer's recommended protocol. IHC for estrogen receptor (ER) and human epidermal growth factor receptor 2 (HER2) were scored according to expression guidelines published by the updated College of American Pathologists (CAP) and the American Society of Clinical Oncology [12], which was recently updated in 2019 [13]. ER and progesterone receptor (PR) were considered negative when under 1%, low positive when 1–10% of tumor nuclei showed staining, and positive when greater than 10% of tumor nuclei showed staining. HER2 was reported as negative if scored as 1+ and positive when scored as 3+. Tumors scored as 2+ underwent confirmatory testing with chromogenic *in situ* hybridization. Positivity for CHGA, SYN, and INSM1 was defined as >10% in tumor cells. IHC for p53 was evaluated in three categories: (1) >5% and <95% weakly stained nuclei wild type (WT), (2) 95% positively and strongly stained nuclei (aberrant p53; missense mutation – MM type), (3) ≤5% weakly stained nuclei (aberrant p53, nonsense mutation – NM type); the MM and NM staining patterns correlate with the presence of *TP53* MM and NM respectively [14]. Rb protein expression by IHC was defined as intact when ≥1% of tumor cell nuclei stained for Rb [11].

### Comprehensive genomic profiling

Comprehensive genomic profiling was performed using the FDA‐approved FoundationOne®CDx assay (Foundation Medicine, Cambridge, MA, USA) in a Clinical Laboratory Improvement Amendments (CLIA)‐certified, CAP‐accredited laboratory from methodologies previously described [15]. Hematoxylin and eosin‐stained slides were reviewed to confirm the presence of tumor followed by nucleic acid extraction. DNA extracted from formalin‐fixed paraffin‐embedded tissues underwent hybrid‐capture based next generation sequencing (NGS) using the FoundationOne platform which interrogates all coding exons of 324 cancer‐related genes and introns from 31 genes commonly rearranged in cancer. Data were analyzed for *TP53* and *RB1* genomic alterations (GAs), including base substitutions, insertions/deletions, copy number alterations, and gene rearrangements.

### Statistical analysis

The Fisher's exact test was used to determine differences in proportions and *t*‐tests were used to compare differences in means for parameters. All tests were two‐sided. These analyses were performed on GraphPad Prism 9.3.0 (345) Serial number: GPS‐2276710 (San Diego, CA, USA). Statistical analysis for TCGA data took place on CBIO portal. mRNA expression *z*‐scores were compared with normal samples with a cutoff of two‐standard deviations to be considered altered. A *p* value <0.05 was considered statistically significant.

## Results

### General information

Seventy‐three cases were collected following review; all were female patients. This cohort contained 68 TNBC, and 5 ER‐low positive cases (1–10% with weak ER nuclear staining) with high Nottingham grade (III/III) [12]. All the cases were PR and HER2 negative. The average age was 59.9 years. Sixty‐one were invasive ductal carcinoma (IDC) not otherwise specified (NOS), five were IDC with apocrine feature, five were IDCs with solid papillary feature, one was SCNEC, and one was IDC with histiocytoid features. Forty‐three cases were negative for CHGA, while one (2%) was positive. Twenty‐nine cases were not evaluated for CHGA. Sixty‐six cases were negative for SYN, while four (6%) were positive. Three cases were not evaluated for SYN. Thirty‐six were negative for INSM1, while 12 (25%) were positive. Twenty‐five cases were not evaluated for INSM1. Out of all cases only 12 cases (16%) had positivity for any NE markers (SYN/CHGA/INSM1). Eleven (15%) of these we consider TNBC‐NED with histologic type as follows: eight IDC NOS, two IDC with apocrine features, and one IDC with solid papillary features. We also identified one separate SCNEC, which had p53/Rb co‐aberrant staining/protein loss.

### TNBC‐NED across different molecular phenotypes

Seventy‐two cases of TNBC‐NED were included after exclusion of the SCNEC. All cases were evaluated by IHC for p53 and Rb. Forty‐four cases (61%) had aberrant staining for p53, and 28 (39%) did not. Rb protein loss was seen in 11 (15%) cases, while Rb was intact in in 61 (85%) cases. p53 aberrant staining molecular correlations by IHC included 26 MM type and 18 NM type. Two cases also had corresponding NGS data by Foundation Medicine. Mutations by NGS included *TP53* loss of exons 2–9, *PTEN* G132V, *ERBB3* H1047R, and *BRCA2* loss in the one case. In the second case, mutations included *TP53* loss of exons 2–9, *PIK3CA H1047R*, *ERBB3 G284R*, *PTEN G132V*, and *RB1* deletion exon 6. In the 61 NE marker negative cases, 37 (61%) cases showed p53 aberrant staining, and 5 (8%) showed Rb protein loss by IHC.

In the 11 TNBC‐NED cases, 4 cases (36%) showed normal p53 staining, while 7 (64%) demonstrated p53 aberrant staining, 5 (45%) cases showed intact Rb, and 6 (55%) had Rb protein loss. Out of the seven TNBC‐NED cases with p53 aberrant staining, three were MM type and four NM type. The seven patients with loss of Rb protein expression all had p53 aberrant staining (co‐aberrant staining/protein loss).

The Ki‐67 proliferative index was 68% in TNBC‐NED compared with 59% in the control group. Relative to other BC, TNBC‐NED seems to be driven by Rb loss (*p* < 0.001), as well as p53/Rb co‐aberrant staining/protein loss (*p* < 0.001). The highest significance was seen in the combined cohort (SYN/CHGA/INSM1), followed by INSM1 alone. SYN positivity did not correlate with any features. A higher mean Ki‐67 proliferative index was associated with p53 aberrant staining (*p* < 0.001), along with Rb loss (*p* < 0.001), and p53/Rb co‐aberrant staining/protein loss (*p* < 0.001). The results from these analyses can be found in Table 1.

**Table 1:** TNBC‐NED across different molecular phenotypes

	CHGA/SYN/INSM1		*P* value	SYN		*P* value	INSM1		*P* value	Ki‐67	*P* value
	+	−		+	−		+	−		%	
p53 aberrant staining (mutation correlate)			0.852			0.289			0.853		**<0.001**
Yes	7	37		1	42		7	24		69	
No	4	24		2	24		4	12		47	
p53 aberrant staining (mutation corelate)			0.341			–			0.173		0.150
MM	3	23		0	26		3	17		72	
NM	4	14		1	16		4	7		62	
Rb protein			**<0.001**			0.400			**0.005**		**<0.001**
Loss	6	5		1	10		6	5		85	
Intact	5	56		2	56		5	31		56	
p53/Rb co‐aberrant staining/protein loss			**<0.001**			0.343			**0.002**		**<0.001**
Yes	6	4		1	9		6	4		85	
No	5	57		2	57		5	32		57	
Ki‐67 (%)	68	59	0.283	60	61	0.965	68	58	0.247	–	–

Significant *p* values are in bold font.

### TNBC‐NED across different clinical and pathological features

Thirty‐one patients received neoadjuvant chemotherapy (NAC), 28 did not, and 13 were status unknown. Pathologic complete response (pCR) was achieved in 10 of 20 patients who had completed NAC and had pathological evaluation of post‐NAC surgical specimens. The post‐NAC stage of those 20 patients was: 9 pT0, 1 pTis, 24 pT1, 9 pT2, 2 pT3, and 6 pT4. Forty‐seven patients received chemotherapy at some point during their cancer treatment (neoadjuvant and adjuvant). Fifty‐four patients received radiation therapy, 3 patients did not, and 15 patients were undocumented. Ten patients had metastatic disease, 58 were negative for metastatic disease, and 4 patients were undocumented. No recurrences were seen, while seven disease specific deaths were documented. The average clinical follow‐up time was 14.4 months. NAC was more commonly seen in cases positive for NE markers: CHGA/SYN/INSM1 (*p* = 0.011), INSM1 (*p* = 0.030). The remaining variables were not found to correlate with NE biomarker status (Table 2). Disease‐specific survival of TNBC‐NED did not differ significantly from other TNBC (hazard ratio = 1.007, *p* = 0.993).

**Table 2:** TNBC‐NED across different clinical and pathological features

	CHGA/SYN/INSM1		*P* value	SYN		*P* value	INSM1		*P* value	Ki‐67	*P* value
	+	−		+	−		+	−		%	−
Age	58	61	0.620	71	60	0.151	58	60	0.702		
NAC			**0.011**			0.999			**0.030**		0.841
Yes	7	24		1	29		7	17		54	
No	0	28		0	26		0	17		54	
pCR			0.158			0.999			0.286		0.662
Yes	1	8		0	10		2	5		53	
No	6	6		0	9		4	2		49	
Stage			0.999			0.999			0.659		0.578
0–1	5	19		0	19		5	10		52	
2–4	2	12		0	12		2	8		57	
Nodal metastasis			0.999			–			0.402		0.894
Yes	1	5		**–**	5		1	1		54	
No	5	34		**–**	37		5	20		52	
Chemotherapy			0.999			0.999			0.999		0.774
Yes	6	40		0	44		6	23		55	
No	1	10		0	10		1	5		53	
Radiation			0.999			0.999			0.999		0.368
Yes	7	46		0	50		7	25		56	
No	0	4		0	4		0	3		46	
Recurrence			0.999			0.999			0.999		–
Yes	0	0		0	0		0	0		–	
No	8	60		1	64		9	36		56	
DFS			0.827			0.999			0.556		0.675
Yes	7	54		1	57		7	33		59	
No	1	6		0	7		1	3		55	
Metastasis			0.851			0.999			0.999		0.494
Yes	1	9		0	10		1	5		60	
No	7	51		1	54		7	31		54	

Significant *p* values are in bold font.

DFS, disease‐free survival.

### TCGA analysis for breast basal/TNBC

**Figure 2:**
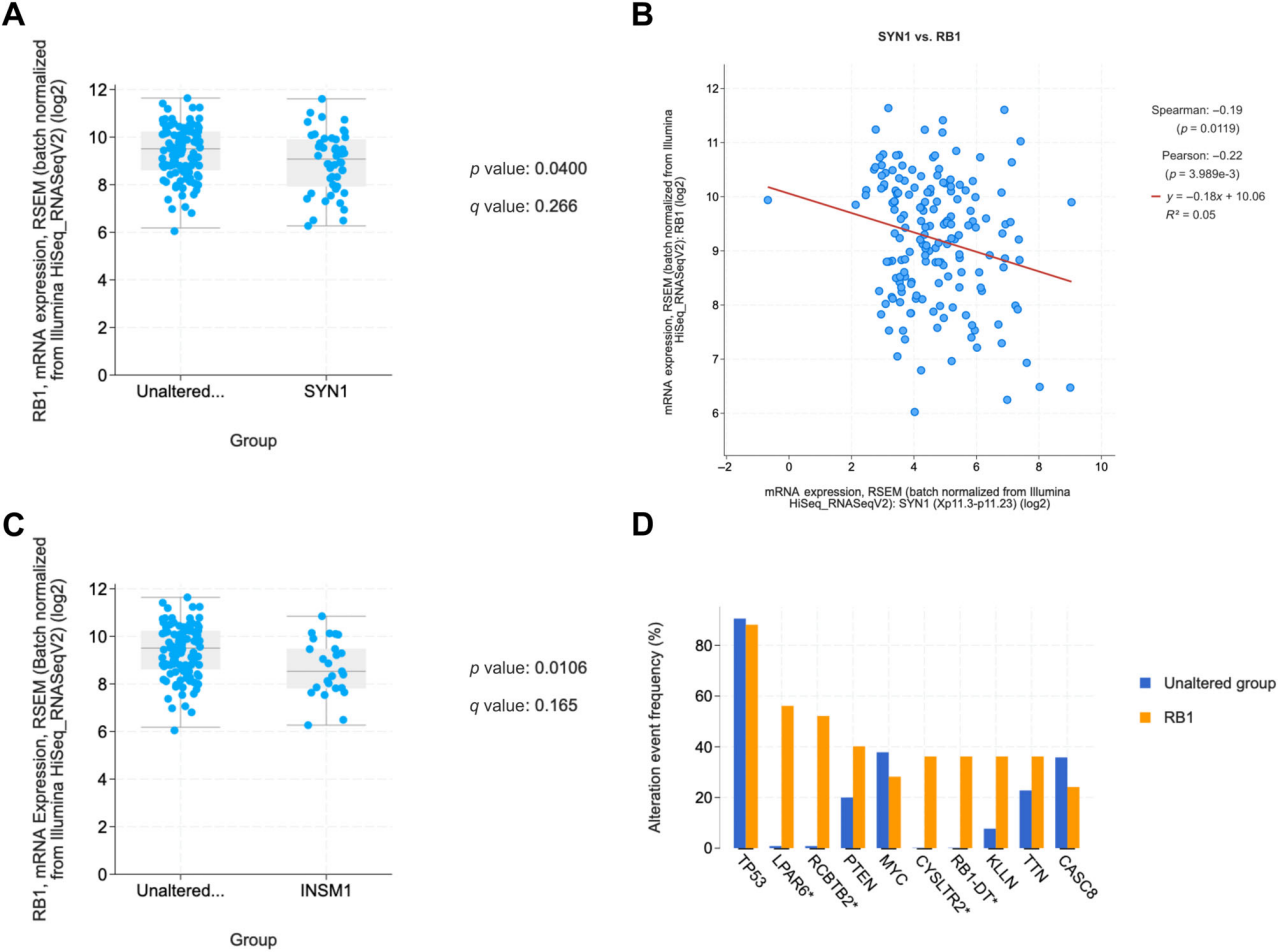
TCGA analysis for breast basal/TNBC. (A) *t*‐Test demonstrating lower *RB1* mRNA expression in tumors with altered SYN1. (B) Spearman and Pearson correlation coefficients between *SYN1* and *RB1* expression. (C) *t*‐Test demonstrating lower *RB1* mRNA expression in tumors with altered *INSM1*. (D) Mutational co‐alteration frequency for *RB1* mutated TNBC‐NED cases.

Analysis of TCGA PanCancer Atlas revealed 171 basal/TNBC patients. Forty‐eight cases (32%) had elevated *SYN1* mRNA expression, 4 cases (2%) had elevated mRNA *CHGA* expression, and 25 cases (15%) had elevated mRNA *INSM1* expression. *RB1* mRNA was abnormal in 34% of cases, and *TP53* mRNA in 51% of cases. Of the 171 TNBC patients, 154 patients had a GA in *TP53* (90%) with a total of 160 driver mutations: 82 MM (51%), 60 truncating (38%), 15 splice (9%), 2 inframe (1%), and 1 fusion (1%). One copy number variation (CNV) was seen for *TP53*, a deep deletion. Twenty‐five patients had a GA in *RB1* (15%) with 7 driver mutations: 6 truncating and 1 fusion, along with 19 CNV, all deep deletions. *RB1* mutational status tended to be more commonly seen in tumors with increased *SYN1* mRNA expression 11 (23%) versus control 18 (15%), although this was not significant (*p* = 0.143). *TP53* mutational status was seen in 43 (89%) cases with increased *SYN1* expression and 111 (90%) cases with normal *SYN1* expression (*p* = 0.467). Increased *INSM1* mRNA expression was seen in 5 (20%) cases with *RB1* mutations versus 24 (16%) cases with normal *INSM1* protein expression, which had *RB1* mutations (*p* = 0.423). *TP53* mutational status was seen in 22 (88%) cases with increased *INSM1* expression and 132 (90%) cases with normal *INSM1* expression (*p* = 0.467). Overall, *RB1* and *TP53* mutational status was not found to be associated with *SYN1*, *CHGA*, or *INSM1* mRNA expression. mRNA expression of *RB1* was correlated negatively with *SYN1* mRNA expression (*p* = 0.0400) and *INSM1* mRNA expression (*p* = 0.0106) in this TNBC cohort (Figure 2). TNBC‐NED defined by increased mRNA expression in any of the three NE markers showed no significant differences in clinical survival compared with TNBC without NED. TNBC‐NED had a higher fraction of genome altered (median = 0.54) versus those with normal expression (median = 0.44) (*p* = 2.181e−3). There was no association between different combinations of decreased *RB1* with increased *SYN1*, *CHGA*, or *INSM1* by mRNA expression. However, 43 TNBCs with *RB1* loss by mRNA also had either increased *SYN1*, *CHGA*, or *INSM1* mRNA expression. This cohort trended toward better survival disease‐specific survival (hazard ratio = 0.35) although this was not statistically significant (*p* = 0.149). No other significant differences in the clinicopathological profile were seen.

## Discussion

Primary tumors of the breast with NED are rare and probably under‐recognized. The overall rate of TNBC‐NED in our cohort was 15% based on positivity of at least one NE IHC marker. In the TCGA cohort, the mRNA expression indicated that *INSM1* is a more sensitive NE marker (38%), followed by *SYN1* (24%) and *CHGA* (6%). However, there are distinctive molecular and immunophenotypic features associated with NED. Our findings suggest the association of Rb loss with positivity for NE markers in TNBC‐NED, a finding found in our internal cohort and validated in the TCGA data set.

This is consistent with the findings from Bean *et al* [10] who examined the clinicopathologic, immunohistochemical, and genetic features of 13 breast NEC including 7 SCNEC, 4 LCNEC, and 2 ANEC. The authors found highly prevalent *TP53/RB1* GAs in 77% (10/13) cases. At the genomic level, *TP53/RB1* co‐alterations were significantly more common in NEC as opposed to grade III IDC‐NST profiled by UCS 500 assay (7/45, 16%). NECs were more frequently Rb negative as compared with a group of 95 grade III IDC‐NST enriched in triple‐negative carcinomas (83% NEC versus 38% IDC‐NST, *p* = 0.004).

In our study, breast tumors from the TCGA cohort with mRNA markers of NED did not show differences in outcomes; however, our analysis was not age and stage matched, and we did not evaluate these tumors for histological features of NED. The association between increased fraction genome alteration (FGA) and increased expression of NED markers is interesting. In certain cancers including the prostate a high FGA is hypothesized to lead to a higher likelihood of aggressive disease due to its impact on cell proliferation and dedifferentiation (cell autonomous) and inhibiting immune responses (noncell autonomous) [16]. Future studies evaluating the significance of FGA in the setting of NE tumor differentiation could be of value.

\The Rb pathway is one of the most studied and is known to be disrupted in numerous ways, including epigenetic silencing, allelic loss, and mutation of *RB1*, among others [17]. Particularly in breast cancer, the role of cyclin D1‐CDK4 kinase activity is important for the phosphorylation of Rb and controlling cell cycle progression. Phosphorylation of Rb results in inactivation and uncontrolled cell proliferation [18]. Looking forward, protein inactivation by phosphorylation could be used to study Rb in breast tumors by kinase activity assays [19], compared with genomic, transcriptomic, and IHC analyses utilized in our study. In the TCGA cohort, *RB1* mutational status did not correlate with mRNA expression of NE biomarkers, unlike *RB1* mRNA expression which did.

Regarding *TP53* and *RB1* molecular alterations and TNBC subtypes, the luminal androgen receptor (LAR), mesenchymal‐like TNBC subtype [20] tumors have been found to be sensitive to CDK4/6 inhibitors due to lower transcriptomic levels of *CCNE1* and *CDK2*, as LAR type TNBCs depend on CDK4/6 to phosphorylate *RB1* to reenter the cell cycle [21]. The basal‐like 1 TNBC subtype is enriched in genes involved in DNA‐damage response and cell‐cycle regulation including *TP53*, amplifications of *MYC*, *CDK6*, or *CCNE1*, along with deletions in *BRCA2*, *PTEN*, *MDM2*, and importantly, *RB1* [21]. Therefore, *TP53* mutational status is prevalent in TNBC [22] and not specific to NEC. However, when combined with Rb protein loss, we found p53 aberrant staining to be significantly associated with positive NE biomarker expression in our internal cohort.

Knudsen *et al* [23] confirmed the aggressive behavior seen in preclinical models of human TNBC with Rb loss showing particularly poor outcomes secondary to MYC overexpression. Breast NENs, along with the expression of different NE biomarkers and Rb loss are under‐recognized broadly because there is currently not well understood outcome or treatment significance. Such differences will be needed to realistically justify the change in practice and pathological classification. Rb loss in TNBC has been found to be associated with higher *CHK1* and *PLK1*, with Rb loss diminishing different checkpoint functions governing DNA replication [24]. This study also demonstrated in xenograft models that Rb loss increased the efficacy of CHK inhibitors. More recently, ^177^Lu‐DOTA‐Evans blue (EB)‐TATE peptide receptor radionuclide therapy was found to be effective at escalating doses in late‐stage NETs [25].

It is important to mention that this was a retrospective study which always introduces the potential for bias. Limitations to this study also include the limited long‐term follow‐up (mean 14.6 months), limited comprehensive molecular data in our cohort, and the lack of immunohistochemical staining in the TCGA cohort. More extensive analysis may better determine the genomic, transcriptomic, and proteomic landscape of TNBC‐NED. It is important to mention that low‐grade TNBC is often NET metastatic to the breast, not breast NEC. Although NET metastasis to the breast is rare, for breast tumors which are triple‐negative, it is important to also consider metastasis such as carcinoid tumors of lung or GI origin.

Five ER‐low positive cases with high‐grade histology were included in this study. ER‐low positive breast cancer is controversial. Although these tumors are not strictly TNBC, data is beginning to suggest they have similar clinical behavior and response to NAC to TNBC [26]. In future, a 10% cutoff may be adopted to define TNBC.

We would like to highlight the importance of these findings and propose TNBC‐NED as an underdiagnosed breast cancer subtype. It is most likely separate from the known spectrum of breast NEN (>90% NE features) and IBC‐NE (≤90% NE features), as these have been primarily based on histology, and routine IHC is not performed in clinical practice. Although morphologically TNBC‐NED overlaps with TNBC‐NST, the unique p53/Rb signature and positivity for INSM1 highlights a genetic and immunophenotypic overlap with NEC of the breast. Long‐term clinical follow‐up studies are needed to better characterize these lesions.

## Author contributions statement

SMH and YW interpreted data, performed study concept and design and acquired the study materials. SMH performed analysis and acquired data. SMH, EY and YW interpreted data, read, revised and approved the final paper.

## Data Availability

All data collected and analyzed in the present study can be made available by the corresponding author upon reasonable request.
